# Impact of initial COVID-19 restrictions on psychiatry presentations to the emergency department of a large academic teaching hospital

**DOI:** 10.1017/ipm.2020.115

**Published:** 2020-09-30

**Authors:** Joseph McAndrew, Julia O’Leary, David Cotter, Mary Cannon, Siobhan MacHale, Kieran C. Murphy, Helen Barry

**Affiliations:** 1 Department of Psychiatry, Beaumont Hospital, Dublin 9, Ireland; 2 Department of Psychiatry, Education and Research Centre, Royal College of Surgeons in Ireland, Dublin, Ireland

**Keywords:** Coronavirus, COVID-19, emergency department, mental health, psychiatry

## Abstract

**Objectives:**

To determine if the initial COVID-19 societal restrictions, introduced in Ireland in March 2020, impacted on the number and nature of psychiatry presentations to the emergency department (ED) of a large academic teaching hospital.

**Methods:**

We examined anonymised clinical data of psychiatry presentations to the ED during the initial 8-week period of COVID-19 restrictions. Data from corresponding 8-week periods in 2018 and 2019 were also extracted for comparison.

**Results:**

Psychiatry presentations to ED reduced by 21% during the COVID-19 restrictions, from 24/week to 19/week when compared with corresponding periods in 2018/2019 (Poisson’s Rate Test estimate of difference −5.2/week, 95% CI 1.3–9.1, *p* = 0.012). Numbers attending for out-of-hours assessment remained unchanged (81 *v*. 80), but numbers seeking assessment during normal hours decreased (71 *v.* 114). We observed increased presentations from the <18 age group, but decreased presentations from the 18 to 29 age group (Pearson’s Chi-Square 20.363, df = 6, *p* = 0.002). We recorded an increase in anxiety disorders during the initial COVID-19 restrictions (31 *v.* 23), and a reduction in alcohol disorders (28 *v.* 52). The proportion of presentations with suicidal ideation (SI) or self-harm as factors remained unchanged.

**Conclusions:**

Rates of emergency presentation with mental illness reduced during the initial COVID-19 restrictions. This may represent an unmet burden of mental health needs. Younger people may be experiencing greater distress and mental illness during the current crisis. More people sought help for anxiety disorders during the COVID-19 restrictions compared with corresponding data from 2018 and 2019.

## Introduction

The COVID-19 pandemic is an ongoing global health emergency caused by the severe acute respiratory syndrome coronavirus 2 (SARS-CoV-2). It emerged in Wuhan, China in late 2019 and has subsequently spread throughout the globe (Shalev & Shapiro, 2020). The World Health Organization (WHO) declared the outbreak a Public Health Emergency of International Concern on 30th January, and a pandemic on 11th March (WHO, 2020a). As of 23rd May, there have been over 5.1 million confirmed cases, and over 333 000 deaths, across 213 countries worldwide (WHO, 2020b). COVID-19 is a viral illness primarily spread by respiratory droplets during close contact. Symptoms include fever, dry cough, malaise, sore throat, new loss of taste or smell, dyspnoea (WHO, 2020c), and in more severe cases can progress to severe acute respiratory syndrome (SARS) and death (Li *et al.* 2020a, 2020b). The estimated fatality rate is 0.5–3%, with approximately 5% of patients requiring critical care support (Shalev & Shapiro, 2020). With no specific treatments or vaccines available for COVID-19, population efforts to combat the disease so far have largely focussed on the prevention of spread via the introduction of public health measures, increased testing capacity within populations, early identification of cases, and robust contact tracing. Public health measures include improved respiratory and hand hygiene etiquette, social distancing measures in public, closure of non-essential economic and recreational activities, restriction of population movements, and application of quarantine and isolation to confirmed or suspected cases (Centers for Disease Control and Prevention (CDC), 2020; Department of Health, 2020; WHO, 2020d, 2020e).

The 2002–2004 SARS epidemic provides the nearest comparable data on the potential impact the current COVID-19 pandemic may have on mental health. Early studies on the psychological morbidity of SARS recorded significantly higher levels of distress amongst affected healthcare workers (Chong *et al*. 2004; Wong *et al.* 2005). Short-to-medium-term studies demonstrated significant levels of anxiety disorders, post-traumatic stress disorder (PTSD), and depression, amongst both healthcare workers and members of the public impacted by SARS (Bai *et al.* 2004; Chan & Huak, 2004; Ko *et al.* 2006; Lee *et al*. 2007; Sim *et al*. 2004). Examination of suicide data from Hong Kong documented a spike in completed suicides amongst older adults in the immediate aftermath of the epidemic (Chan *et al*. 2006; Cheung *et al.* 2008).

The first case of COVID-19 was reported in Ireland on 29th February. From late February until 12th March, the Department of Health considered Ireland to be in the ‘Containment Phase’ of its pandemic response. On 12th March, the Government of Ireland commenced the phased introduction of increasingly restrictive public health measures designed to combat the spread of COVID-19, heralding the beginning of the ‘Delay Phase’ of the pandemic response. Initially, this involved the closure of all childcare facilities, schools, colleges, and cultural institutions. People were directed to work from home where possible, and large indoor and outdoor gatherings were directed to be cancelled. On 27th March, the government instructed all non-essential businesses, recreational, and economic activities to cease. All public gatherings were prohibited, non-essential travel was banned, and people were instructed to stay indoors except for specific purposes such as essential shopping, healthcare needs, and limited exercise within a 2 km distance from home. Higher risk groups such as people aged over 70 were advised to cocoon (self-isolate) at home until further notice. Social distancing measures were to be strictly adhered to in public, and the Garda Siochana were granted unprecedented legal powers to ensure compliance with the measures. The so-called ‘Lockdown’ was extended further on 10th April. Since early May, the government has begun to slowly reverse the COVID-19 societal restrictions; by allowing people to travel up to 5 km from their home, and by permitting those who are cocooning to leave home for exercise, provided they avoid contact with all others (Government of Ireland, 2020).

Beaumont Hospital (BH) is a large academic teaching hospital 5 km north of Dublin City centre. With 3000 staff and 820 beds, it provides emergency, acute care, elective, and tertiary services across 54 medical specialties to a local community of some 290 000 people (Beaumont Hospital, 2020). From May 2014, with the opening of an acute psychiatric inpatient unit on the hospital campus, BH emergency department (ED) became the site of crisis mental health assessments for the local catchment area. We wanted to determine if the initial COVID-19 societal restrictions, introduced in Ireland in March 2020, had impacted on the number and nature of psychiatry presentations to the ED, when compared with available data from 2018/2019.

## Methods

BH Liaison Psychiatry Department uses an Electronic Patient Record (EPR) to record clinical, demographic, and attendance details for all psychiatry presentations to the ED. Diver 7, a business intelligence data analysis and reporting tool, was used to extract anonymised data from the psychiatry EPR clinical database, in relation to psychiatry presentations to the ED during the initial 8-week period of COVID-19 restrictions in Ireland (16th March 2020–10th May 2020). Data from corresponding 8-week periods in 2018 (19th March 2018–13th May 2018) and 2019 (18th March 2019–12th May 2019) were also extracted for comparison, as well as data on weekly ED psychiatry presentation rates throughout the entirety of 2018 and 2019.

Anonymised data were stored in a Microsoft Excel Worksheet. Basic data processing was undertaken using Microsoft Excel. Statistical analysis was performed using the Minitab 17 Statistical Software Package. A Poisson’s Process was used to model the rate of both psychiatry presentations and total presentations to the ED. The Poisson’s Rate Test was used to test for significant differences in the weekly rate of psychiatry presentations to ED, and the weekly rate of total presentations to ED during the COVID-19 restrictions *versus* 2018/2019. The 2-sample Proportion Test was used to test for significant differences in psychiatry presentations as a proportion of total ED presentations, self-harm or suicidal ideation (SI) as proportions of weekly psychiatry presentations, anxiety disorders as a proportion of total psychiatry presentations, and alcohol disorders as a proportion of total psychiatry presentations, during the COVID-19 restrictions *versus* 2018/2019. The Pearson’s Chi-Square test was used to test for any significant difference in the distribution of psychiatry presentations to the ED by age group, during the COVID-19 restrictions *versus* 2018/2019.

## Results

**Fig. 1.**
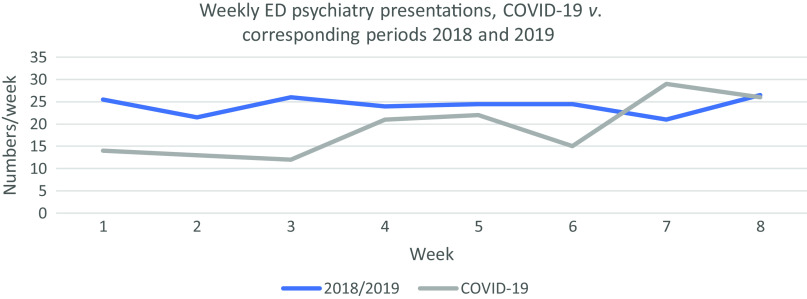
Weekly ED psychiatry presentations during the initial 8 weeks of COVID-19 restrictions *versus* the corresponding periods from 2018/2019.

During the initial 8 weeks of COVID-19 restrictions, we recorded a mean of 19 (±6.4) psychiatry presentations to the ED per week, compared with 24 (±3.2)/week in the corresponding period from 2018/2019. This represents a statistically significant 21% reduction in the weekly rate of psychiatry presentations to ED (Poisson’s Rate Test estimate of difference −5.2/week, 95% CI 1.3–9.1, *p* = 0.012). See Fig. 1.

We also observed a 30% reduction in the rate of total ED presentations (all specialities) from 1131/week (±43.9) to 789/week (±139.3) (Poisson’s Rate Test estimated difference −341.7, 95% CI 316.2–367.2, *p* < 0.001). As a percentage of total ED presentations, psychiatry normally accounts for 2.1% (±0.3%), but this increased to 2.4% (±0.6%) during the initial COVID-19 restrictions. This relative proportional increase did not reach statistical significance (2-sample Proportion Test, *p* = 0.224), but as the absolute proportions are so small relative to total ED presentations, it is possible that any significance was obscured.

**Fig. 2.**
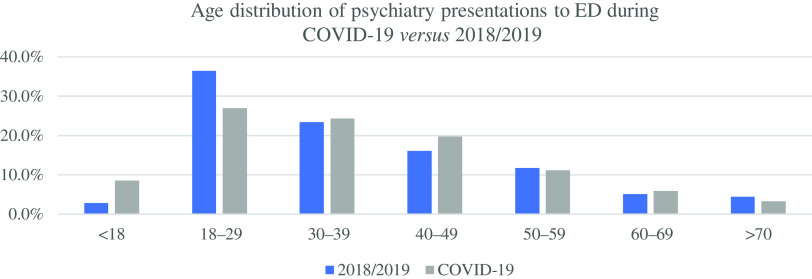
Age distribution of psychiatry presentations to ED, during the initial 8 weeks of COVID-19 restrictions *versus* 2018/2019.

A significant difference in the age distribution of psychiatry presentations to the ED during the COVID-19 restrictions was observed when compared against the age distribution of all psychiatry presentations to the ED throughout 2018/2019 (Pearson’s Chi-Square 20.363, df = 6, *p* = 0.002). The greatest departures from independence were observed in the <18 and 18–29 age groups. There were increased presentations from the <18 age group (13 *v.* 2) and decreased presentations from the 18 to 29 (41 *v.* 62) age group. See Results in Table 1 and Fig. 2.

**Table 1.** Distribution of psychiatry presentations by age 2018/2019 *v.* 2020

Age group	2018/2019 (mean)	2020 (mean)	
<18	2	13	Pearson’s Chi-Square test statistic = 20.363 df = 6 *p*-value = 0.002
18–29	62	41	
30–39	54	37	
40–49	28	30	
50–59	26	17	
60–69	13	9	
>70	10	5	
Time of psychiatry presentations 2018/2019 *v.* 2020			
Normal hours	114	71	2-sample Proportion Test estimated difference = 0.12 (95% CI 0.03–0.22) *p*-value = 0.012
On-call hours	80	81	

**Fig. 3.**
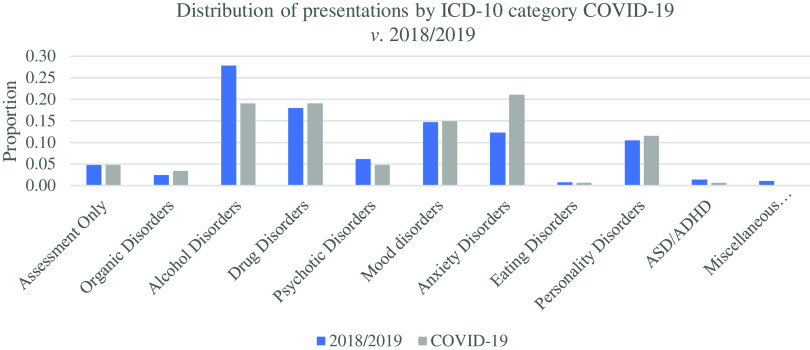
Distribution of psychiatry presentations to the ED by ICD-10 diagnostic category, during the initial 8 weeks of COVID-19 restrictions *versus* corresponding periods from 2018/2019.

We recorded differences in the distribution of psychiatry presentations to the ED by ICD-10 diagnostic category, during COVID-19 restrictions *versus* corresponding periods 2018/2019. We stratified presentations to the following categories for our analysis; Assessment only (Z00.4), Organic Disorders (F00 group), Alcohol Disorders, Drug Disorders, Psychotic Disorders (F20 group), Mood Disorders (F30 group), Anxiety Disorders (F40 group), Eating Disorders, Personality Disorders, ASD/ADHD, Miscellaneous Disorders. We subdivided the F10 diagnostic category into Alcohol Disorders and Drug Disorders, as we felt this would be more informative. There were 31 presentations with anxiety disorders during the COVID-19 restrictions, compared to a mean of 23 during the corresponding periods in 2018/2019. This was reflected in a significant proportional increase in Anxiety Disorders during the initial COVID-19 restrictions *versus* the corresponding periods from 2018/2019 (2-sample Proportion Test, estimate for difference +0.085, 95% CI 0.013–0.157, *p* = 0.020). Conversely, the proportion of psychiatry presentations to the ED with an Alcohol Disorder significantly reduced during the initial COVID-19 restrictions when compared with the corresponding periods from 2018/2019 (2-sample Proportion Test, estimate for difference −0.085, 95% CI 0.009–0.160, *p* = 0.029). There were 28 presentations with alcohol as a factor, down from a mean of 52 in the corresponding 2018/2019 period. Significant differences were not observed within the other diagnostic categories. See Results in Table 2 and Fig. 3.

**Table 2.** Distribution of psychiatry presentations by ICD-10 category 2018/2019 *v.* 2020

	2018/2019 (mean)	2018/2019 (proportion)	2020 (mean)	2020 (proportion)	Estimated difference (2-sample Proportion Test)	*p*-value
Assessment only (Z00.4)	9	0.05	7	0.05	0.00	0.975
Organic Disorders (F00 group)	5	0.02	5	0.03	0.01	0.559
Alcohol Disorders	52	0.28	28	0.19	0.09	0.027
Drug Disorders	34	0.18	28	0.19	−0.01	0.775
Psychotic Disorders (F20 group)	12	0.06	7	0.05	0.01	0.514
Mood Disorders (F30 group)	28	0.15	22	0.15	−0.00	0.949
Anxiety Disorders (F40 group)	23	0.12	31	0.21	−0.09	0.020
Eating Disorders	2	0.01	1	0.01	0.00	0.880
Personality Disorders	20	0.10	17	0.12	−0.01	0.719
ASD/ADHD	3	0.01	1	0.01	0.01	0.464
Miscellaneous Disorders	2	0.01	0	0.00	0.01	0.044

Presentations during normal working hours were reduced from a mean of 114 presentations in 2018/2019 to 71 presentations during the COVID-19 restrictions. Number of on-call, or out-of-hours presentations were similar, with 81 presenting during the COVID-19 period for assessment *versus* a mean of 80 during the comparative periods 2018/2019. See Results in Table 1.

During the COVID-19 restrictions, the mean rate of SI presentations was 11/week (±4.6) *versus* 13/week (±3.5) for the corresponding period in 2018/2019, with no significant difference observed (Poisson’s Rate Test, *p* = 0.290). The mean rate of self-harm presentations during the COVID-19 restrictions was 7/week (±4.1), compared with 8/week (±2.1) in 2018/2019, with no significant difference observed (Poisson’s Rate Test, *p* = 0.242).

## Discussion

We observed that the weekly rate of psychiatry presentations to the ED significantly reduced during the initial COVID-19 restrictions. Were less people experiencing a mental health crises or was fear of the hospital environment preventing presentation? Early evidence on psychiatric morbidity from China indicates that the rates of mental illness during the COVID-19 pandemic may be expected to rise rather than fall. For example, web-based surveys of mental health in China suggest significant levels of psychological distress secondary to the COVID-19 pandemic at a population level (Wang *et al*. 2020). Factors identified included concerns secondary to media reports, and perceived impacts of the viral pandemic. A content analysis of Weibo posts (a social media platform in China) from 17 865 active online users in China during the pandemic revealed a significant increase in negative emotional content and sensitivity to perceived social risks (Wang *et al*. 2020). A further web-based study from the COVID-19 pandemic in China suggests an increased prevalence of generalised anxiety disorder (GAD), depressive symptoms, and an overall reduction in sleep quality (Huang & Zhao, 2020). It identified younger people and healthcare workers as being more vulnerable to these conditions, and to the psychological stress secondary to the outbreak. Another Chinese study confirmed increased psychological distress in the population, identifying minors and healthcare workers as higher risk groups (Tian *et al*. 2020). Shinn & Viron (2020) highlighted that people with serious mental illness may be disproportionately distressed by social distancing and enforced isolation measures. Furthermore, cognitive deficits, executive dysfunction, and negative health-related behaviours within this group may put them at a greater risk of both COVID-19 infection, and more adverse outcome (Percudani *et al*. 2020; Shinn & Viron, 2020).

If psychiatric morbidity in the population can be expected to increase during a pandemic, then we can hypothesise as to the reasons behind the observed decrease in attendance at ED. During the COVID-19 restrictions, the number of out-of-hours psychiatric presentations remained largely unchanged, but the numbers of ‘normal hours’ presentations reduced from 114 to 71. Perhaps a proportion of the ‘normal hours’ presentations pre COVID-19 did not actually require emergency psychiatric care, or perhaps people have intentionally avoided attending ED during the initial COVID-19 restrictions due to fear of contracting COVID-19 from the ED environment itself? Have these people accessed care and support from elsewhere, such as primary care, community mental health teams, or the voluntary sector? If people have not been accessing mental health support from alternative sources, then this potentially represents a significant burden of unmet mental health needs within the population. Overall rates of attendance at ED for any reason were significantly reduced by approximately 30% during the initial COVID-19 restrictions, and we postulate that it was perhaps fear of contracting COVID-19 that acted as a deterrent for many of these people. In tandem with this reduction, the rate of psychiatry presentations to the ED also decreased, but only by 21%.

A number of studies have documented a significant increase in completed suicides amongst older people in Hong Kong in the year following the SARS outbreak of 2003/2004 (Chan *et al*. 2006; Cheung *et al*. 2008). In our data, the rate or proportion of presentations with either SI or self-harm as a factor during the initial COVID-19 restrictions did not differ significantly when compared with data from 2018/2019, but this may reflect less people seeking assistance via ED in these circumstances. Kawohl and Nordt, in The Lancet, recently modelled the effect of unemployment on suicide on the basis of global public data from 63 countries, and observed that suicide risk was elevated by 20–30% when associated with unemployment during 2000–2011 (including the 2008 economic crisis). Using this data to analyse the effects of the current pandemic-associated worldwide recession, they predict that suicides may increase by almost 10 000 per year (Kawohl & Nordt, 2020). Whether suicide rates in Ireland change during the initial phase of COVID-19 restrictions remains to be seen.

Given mass school closures, uncertainty around official state exams due to take place in the summer, cancellation of most recreational and social activities, and prolonged social distancing measures, we were unsurprised that the number of psychiatry presentations to the ED represented by young people <18 years old, increased from a mean of 2 presentations in the 2018/2019 period to 13 presentations during the COVID-19 restrictions. Conversely, in the 18–29 age group, we recorded reduced numbers of psychiatric presentations during the COVID-19 restrictions *versus* corresponding periods 2018/2019 (41 *v.* 62). The reasons for this are unclear. Perhaps this group had reduced mental health needs during the pandemic, were reluctant to attend ED for fear of COVID-19 infection, or maybe they were seeking mental health support elsewhere?

When analysing the differences in the distribution of psychiatry presentations to the ED by ICD-10 diagnostic category, we observed an increase in anxiety disorders during the initial COVID-19 restrictions. Early data from an ongoing study at the National University of Ireland Galway (NUIG) also suggests significantly higher levels of self-reported anxiety levels amongst respondents (NUIG, 2020). This is consistent with the early studies from China referenced above and is in line with long-term follow-up studies following the SARS epidemic in 2003/2004. Lam *et al.* examined the longer term psychiatric morbidity in SARS survivors and found that over 40% of participants had an active psychiatric diagnosis 2–3 years later (compared with 3% prior to contracting SARS). The most common diagnoses identified were PTSD, somatoform pain disorder, panic disorder, and obsessive-compulsive disorder (Lam *et al.* 2009). Another long-term study on the psychiatric morbidity post-SARS by Mak *et al.* (2009) reported a post-SARS cumulative incidence of DSM-IV psychiatric disorders of 58.9%. The most common diagnoses were PTSD, depression, and anxiety spectrum disorders.

We observed far less patients presenting with Alcohol Disorders during the initial COVID-19 restrictions when compared against the corresponding periods from 2018/2019. It’s unclear if all alcohol-related presentations to ED decreased during this period, or if this represents relatively less cases being referred to with psychiatric morbidity. Our results contrast with a recent survey by the Central Statistics Office (CSO) examining consumption habits as part of the social impact of COVID-19; 22.2% of respondents reported increased alcohol consumption, and these changes were most pronounced in the 18–44 age range (CSO, 2020). Revenue, in their quarterly national alcohol clearance figures, showed reduced sales of alcohol overall in the first two quarters this year but increased levels of wine and spirit consumption despite the mass closure of pubs and restaurants (Revenue Commissioners, 2020). We speculate that this may represent a shift towards greater levels of alcohol consumption at home, and perhaps may have led to a reduction in people being brought to the ED by ambulance or by the Gardai (after having been found intoxicated in public places).

## Conclusion

Our study shows that weekly psychiatry presentations to the ED fell by approximately 21% during the initial 8 weeks of COVID-19 restrictions, with the observed reduction largely due to reduced attendances during ‘normal hours’. Whilst this may be a reflection of reduced psychiatric morbidity we feel it is more likely a consequence of people avoiding ED due to fear of contracting COVID-19, and as such may represent an unmet burden of mental ill-health in the population. Despite the overall reduction, more people from the <18 age group sought assistance with their mental health via the ED, but there was a decrease in presentations from the 18 to 29 age group. We observed an increase in anxiety-related presentations, but a reduction was seen in alcohol-related psychiatric presentations. The latter may be reflective of decreased total sales of alcohol during the initial COVID-19 restrictions, or may represent a shift towards greater levels of alcohol consumption at home, with decreased incidents of intoxication in public, and reduced numbers of people being brought to the ED intoxicated, by ambulance or by the Gardai. Further studies to explore these patterns of emergency psychiatry presentations during the COVID-19 pandemic would be helpful to identify high-risk groups not seeking help, and may aid with mental health service delivery for the remainder of the pandemic.
